# A Low-Cost High-Performance Data Augmentation for Deep Learning-Based Skin Lesion Classification

**DOI:** 10.34133/2022/9765307

**Published:** 2022-04-26

**Authors:** Shuwei Shen, Mengjuan Xu, Fan Zhang, Pengfei Shao, Honghong Liu, Liang Xu, Chi Zhang, Peng Liu, Peng Yao, Ronald X. Xu

**Affiliations:** ^1^First Affiliated Hospital, University of Science and Technology of China, Hefei 230031, China; ^2^Suzhou Institute for Advanced Research, University of Science and Technology of China, Suzhou 215000, China; ^3^Department of Precision Machinery and Precision Instrumentation, University of Science and Technology of China, Hefei 230026, China

## Abstract

*Objective and Impact Statement*. There is a need to develop high-performance and low-cost data augmentation strategies for intelligent skin cancer screening devices that can be deployed in rural or underdeveloped communities. The proposed strategy can not only improve the classification performance of skin lesions but also highlight the potential regions of interest for clinicians’ attention. This strategy can also be implemented in a broad range of clinical disciplines for early screening and automatic diagnosis of many other diseases in low resource settings. *Methods*. We propose a high-performance data augmentation strategy of search space 10^1^, which can be combined with any model through a plug-and-play mode and search for the best argumentation method for a medical database with low resource cost. *Results*. With EfficientNets as a baseline, the best BACC of HAM10000 is 0.853, outperforming the other published models of “single-model and no-external-database” for ISIC 2018 Lesion Diagnosis Challenge (Task 3). The best average AUC performance on ISIC 2017 achieves 0.909 (±0.015), exceeding most of the ensembling models and those using external datasets. Performance on Derm7pt archives the best BACC of 0.735 (±0.018) ahead of all other related studies. Moreover, the model-based heatmaps generated by Grad-CAM++ verify the accurate selection of lesion features in model judgment, further proving the scientific rationality of model-based diagnosis. *Conclusion*. The proposed data augmentation strategy greatly reduces the computational cost for clinically intelligent diagnosis of skin lesions. It may also facilitate further research in low-cost, portable, and AI-based mobile devices for skin cancer screening and therapeutic guidance.

## 1. Introduction

Skin diseases represent one of the most common health problems globally [1] that affect patients’ quality of life, induce significant socioeconomic burden to society, and even lead to increased morbidity and mortality [2]. Skin cancer is a family of skin diseases caused by the neoplastic growth of skin cells in the epidermis and can be classified into two major categories of nonmelanoma and melanoma [3]. Nonmelanoma skin cancer (NMSC) accounts for 98% of all skin cancers, and their treatment places a significant burden on the healthcare systems [4]. Melanoma accounts for only 2% of all skin cancers but causes the most skin cancer deaths [3]. Early detection and prompt treatment of skin lesions can significantly improve quality of life and reduce melanoma mortality for patients. The previous study has revealed an elevated 5-year survival of 99% for early detected melanoma, in comparison with that of ~18% with late diagnosis [5]. The most commonly used criteria for skin cancer diagnosis is based on visual inspection of lesion size, shape, color, and location [6]. Although using a dermoscope helps to improve the diagnostic accuracy [7], visual inspection represents a subjective method for skin cancer detection, and its accuracy heavily depends on the examiner’s experience. Due to the global shortage in experienced dermatologists, patients in rural communities and low resource settings have experienced the significant delay in detection and treatment of skin cancer as well as the higher morbidity and mortality compared with other areas [1].

To address the shortage of dermatology specialists and improve the accuracy for skin cancer classification, various artificial intelligent (AI) diagnostic technologies have been explored [8]. Since the first report in 1987, traditional machine learning techniques have been applied to help dermatologists in faster data process and more reliable diagnosis [9]. Machine learning algorithms, such as support vector machine, have already achieved a classification accuracy of 80% [10]. However, the performance of these methods in multiclass classification is limited by many deficiencies such as excessive adjustment (i.e., overfitting). With recent advances in computing technology, deep convolutional neural network (DCNN) has been introduced into the skin disease diagnosis and has achieved the encouraging diagnostic accuracies better than 90%, comparable or even superior to those of dermatologists [11].

Although DCNN has been integrated with several mobile dermoscopic devices for intelligent classification of skin lesions [12], rural deployment of DCNN classifiers for skin cancer screening is hindered by multiple technical challenges in computational cost, portability, and reliability. It has been revealed that DCNN classifiers tend to work well when they are trained on large datasets acquired from actual clinical cases [13]. However, most of the publically accessible skin lesion imaging depositories, such as PH^2^ (160 nevi and 40 melanomas), Derm7pt (1011 lesion cases, and the number of SC and BCC is 45 and 42), ISIC 2017 (13768 images, and the number of AKIEC is only 2), ViDIR series (<4,000 images with partial pathologic verification), and HAM10000 (10015 images in seven skin disease categories) [14–16], have either a relatively small data size or an uneven distribution of disease types, limiting the achievable performance of the trained classifiers. Similarly, the lack of a large-scale, reliable, and balanced clinical dataset presents a common barrier for developing and deploying high-performance DCNN models in many other clinical disciplines.

Ensembling multiple deep learning (DL) models and data augmentation are commonly used methods to overcome the aforementioned limitations of the available clinical datasets. Model ensembling is a process that aggregates the predictions of multiple diverse models in one final prediction, where the various models are trained using different strategies [17]. This approach improves the prediction performance by reducing the generalization error of the prediction, as evidenced by the observation that the balanced multiclass accuracy of the ensembling models is generally higher than that of the single models in the ISIC 2018 challenge [18]. However, the computational cost of the ensembling models is generally high, and the ceiling effect prevents their further improvement of diagnostic accuracy. Therefore, it is necessary to improve the performance of individual DCNN models. Data augmentation helping increase both “amount” and “diversity” of the existing dataset is one of the keys, and conventional data augmentation strategies include scaling, translation, rotation, random cropping, image mirroring, and color change which have been developed [19]. However, those data augmentation strategies are typically dataset-specific [20]. Recently, the emerging automatic data augmentation techniques, such as AutoAugment [20], Fast AutoAugment [21], Population-Based Augmentation [22], and RandAugment [23], have shown a certain superiority over conventional data augmentation strategies. Nevertheless, our experiments have found that these enhancement methods do not perform well on small medical databases, and a new search is needed. Moreover, the search space sizes of AutoAugment [20], Fast AutoAugment [21], Population-Based Augmentation [22], and RandAugment [23] are 10^32^, 10^32^, 10^61^, and 10^2^, respectively. The high computational costs of both the ensembling models and data augmentation strategy searching are therefore preventing the practical implementation of DCNN-based intelligent diagnosis for skin diseases.

We therefore propose a high-performance data augmentation strategy suitable to be implemented in low resource settings and the potential to further help developing mobile devices for AI-based skin lesion detection. In a two consecutive stages of augmentation search and network match, the best augmentation strategy is first searched in the space of Low-Cost-Augment (LCA) under the specified criteria with 5-fold cross-validation. Then, the DCNN models adopting the searched augmentation strategy are fine-tuned using the full training set, and the one of highest specified criteria is matched as the best combination. In this paper, the efficiency of such a data augmentation strategy is validated on the HAM10000, ISIC 2017, and Derm7pt [15, 16] datasets using the EfficientNet models as a baseline. EfficientNet is a group of lightweight convolutional network models achieving state-of-the-art accuracy with an order of magnitude fewer parameters and FLOPs over ImageNet [24]. In the meantime, the Class Activation Mapping (CAM) liked technology [25], utilizing the gradients of models’ final convolutional layer, can generate a visual and explainable heatmap for the DCNN model. It helps to make the “black box” nature of deep learning more clear and highlights the position and scope of lesions distribution. Therefore, the Grad-CAM++ [25] is introduced to verify the scientific rationality of the proposed method and its potential in promoting the integration of personalized diagnosis and treatment technology.

Our research contributions can be summarized as follows:

(1) We propose an argumentation strategy of search space in 10^1^ magnitudes, which can be combined with any model through a plug-and-play mode, and of the ability to search for the best argumentation method for a medical database with low resources cost
(2) By training the EfficientNet model using the proposed data augmentation strategy on the HAM10000 dataset, we have achieved a BACC level of 0.853, ranking top of the ISIC 2018 challenge in the channel of single-model and no-external-database. The searched method also achieves excellent performance on the ISIC-skin 2017 dataset and Derm7pt dataset
(3) With the combination of the proposed DL model and Grad-CAM++, we can not only achieve high performance on different datasets at a low computation cost but also verify the scientific rationality of diagnostic models and diagnostic models’ potential in promoting the integration of personalized diagnosis and treatment technology

The proposed DL strategy can be also expanded to other clinical disciplines for automatic screening and accurate detection of diseases in areas of limited resources. All the source code used in this paper is available in our public repository (http://github.com/Shuwrood-SSW/Low-cost-and-high-performance-data-augmentation-for-deep-learning-based-skin-lesion-classification).

## 2. Results

### 2.1. Evaluation of Augmentation Strategies in a 5-Fold Cross-Validation on HAM10000 Dataset

Totally, 12 subpolicies in the search space of Low-Cost-Augment are defined. Each subpolicy

comes with 5 uniformly spaced probabilities ([0.1, 0.3, 0.5, 0.7, and 0.9]) and random magnitudes, leading to a search space size of 12∗5=60 possibilities. In comparison, the search space sizes of AutoAugment [20], Fast AutoAugment [21], Population-Based Augmentation [22], and RandAugment [23] are 10^32^, 10^32^, 10^61^, and 10^2^, respectively. Notably, the proposed strategy greatly reduces the search space and thereby decreases the computational costs.

Table 1 shows the BACC performance of the trained EfficientNets after applying the proposed LCA strategy at various probabilities on the HAM10000 dataset. According to the table, the BACC values show a trend of first increasing and then decreasing from EfficientNets b0-b7, and the deviations fluctuate within ±0.033 in 5-fold cross-validation, no matter which augmentation strategy is used. These results imply that the most complex network is not necessarily suitable for the HAM10000 dataset of medium capacity. It is also observed that the data augmentation strategy in the probability of 0.1 or 0.3 generally performs better than that of the other probabilities, as five out of the eight models yield the higher BACC values at the probability of 0.3.

**Table 1:** BACC of EfficientNet b0-b7 trained adopting the LCA strategy in different probabilities (P).

DCNNs	P=0.1	P=0.3	P=0.5	P=0.7	P=0.9
EfficientNet b0	0.881±0.025	0.883±0.014	0.881±0.015	0.874±0.021	0.875±0.024
EfficientNet b1	0.881±0.033	0.882±0.016	0.873±0.020	0.880±0.027	0.873±0.027
EfficientNet b2	0.879±0.019	0.883±0.013	0.881±0.016	0.876±0.024	0.880±0.029
EfficientNet b3	0.873±0.027	0.870±0.016	0.867±0.018	0.868±0.015	0.859±0.019
EfficientNet b4	0.878±0.021	0.876±0.013	0.874±0.019	0.871±0.020	0.873±0.014
EfficientNet b5	0.859±0.032	0.868±0.018	0.858±0.024	0.858±0.023	0.845±0.017
EfficientNet b6	0.871±0.016	0.866±0.012	0.857±0.033	0.862±0.033	0.856±0.033
EfficientNet b7	0.864±0.028	0.865±0.01	0.865±0.014	0.851±0.032	0.848±0.019

**Figure 1:**
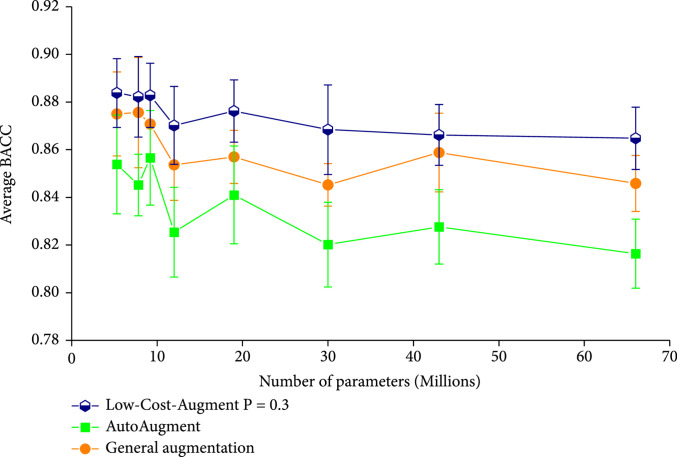
BACC performance of EfficientNets trained adopting the best LCA strategy (n=5), ImageNet-based AutoAugment strategy (n=5), and General Augmentation strategy (n=5). The DCNNs from left to right on the x-axis correspond to EfficientNet b0-b7.

The BACC performance of the LCA strategy at the probability of 0.3 is compared with other augmentation strategies, as shown in Figure 1. According to the figure, the performance of the LCA strategy exceeds both the General Augmentation and the searched AutoAugment strategies. These results indicate that the LCA strategy at the probability of 0.3 effectively reduces the overfitting risk and is more suitable for the HAM10000 dataset. It is also observed from the figure that the General Augmentation strategy performs better than the ImageNet-based AutoAugment strategy, indicating that the augmentation strategy obtained from one dataset cannot be effectively transferred to the other datasets.

### 2.2. Visual Representations of Searched DL Algorithms on the HAM10000 Dataset

EfficientNet b0-b7 are finely tuned by adopting the two-stage data augmentation strategy at the probability of 0.3 on the HAM10000 full dataset. The predicted results of the test dataset on the fine-tuned models at different epochs are uploaded to the official website of ISIC 2018 Challenge in order to obtain the BACC values and other performance metrics. Based on our experience, the BACC value for the models of over 30 epochs only fluctuates within a small range; therefore, we collect the official test results in the range of 30~90 epochs with 5 epoch intervals.

**Figure 2:**
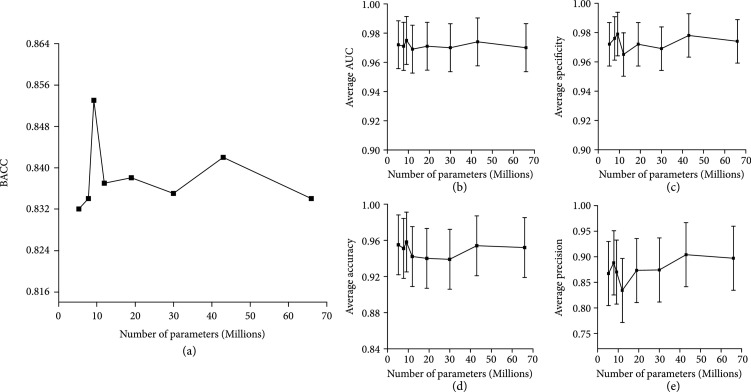
Performance of (a) BACC, (b) average AUC (n=7), (c) average specificity (n=7), (d) average accuracy (n=7), and (e) average precision (n=7) of EfficientNets trained adopting the searched augmentation strategy. The DCNNs from left to right on the x-axis correspond to EfficientNet b0-b7.

Figure 2 shows the best official performance of the EfficientNet models trained by the proposed LCA strategy. Although no obvious correlation is observed between the optimal BACC value and the corresponding parameters of different EfficientNet models, the EfficientNet b2 achieves the highest BACC value of 0.853, better than any other models. Similar trends are also observed in other metrics of the figure, such as the average area under the receiver operating characteristic curve (AUC), the average accuracy, the average specificity, and the average precision. Further analysis of the EfficientNet b2 performance in seven classes of HAM10000 (Table S4) indicates that the diagnostic specificity of the model is greater than 0.983 for all the classes and the diagnostic accuracy is greater than 0.91 for all the classes except NV.

The performance of our augmentation strategy is also compared with those on the ISIC 2018 Challenge legacy leaderboard, as listed in Table 2. According to the table, using ensembling multiple models or using external datasets typically achieve better performance, such as those by Minjie, MetaOptima Technology Inc. [18], DAISYLab [26], Amirreza [27], and IPM-HPC. In the case where only a single model is used without an external database, the BACC reported on the legacy leaderboard (https://challenge.isic-archive.com/leaderboards/2018 (Task 3: Lesion Diagnosis)) typically does not exceed 0.8, and the latest study on the live leaderboard (https://challenge.isic-archive.com/leaderboards/live (2018.3: LESION DIAGNOSIS)) does not exceed 0.836. Therefore, the BACC performance of EfficientNet b2 using our augmentation strategy ranks the first on the channel of “single-model” and “no-external-data,” even better than some of the ensembling models. It is also worth noting that our proposed strategy achieves the melanoma diagnostic sensitivity superior to the ensembling models and those using external datasets.

**Table 2:** The performance of models on ISIC 2018 challenge legacy leaderboard (rows 1–3), live leaderboard (rows 4–11), and our proposed approach.

Team/authors	Use external data	Use ensemble models	BACC	Sensitivity for melanoma	Avg. AUC	Avg. specificity
Nozdryn et al. [18]	Yes	Yes	0.885	0.760	0.983	0.833
Gessert et al. [26]	Yes	Yes	0.856	0.801	0.987	0.984
Zhuang et al.	No	Yes	0.845	0.702	0.978	0.980
Minjie	Yes	Yes	0.895	0.778	0.982	0.981
Amirreza et al.	Yes	Yes	0.874	0.585	0.979	0.992
IPM_HPC.	Yes	Yes	0.866	0.830	0.976	0.976
Amirreza et al. [27]	Yes	Yes	0.862	—	0.981	—
Mahbod et al.	No	No	0.836	0.719	0.975	0.982
ND Reddy et al.	No	No	0.735	0.544	0.945	0.968
Our approach	No	No	0.853	0.789	0.975	0.973

### 2.3. Generality Verification of the Searched Method on ISIC 2017 and Derm7pt Datasets

**Figure 3:**
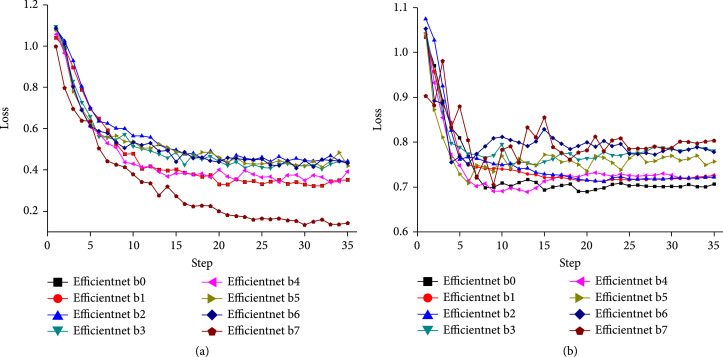
Loss curve of (a) train data and (b) test data on ISIC 2017 dataset. Here, the DCNNs from left to right on the x-axis correspond to EfficientNet b0-b7.

To verify the generality of the searched method, HAM10000-pretrained EfficientNet b0-b7 of performance in Figure 3 are furthermore fine-tuned by adopting the LCA augmentation in 0.3 probability on ISIC 2017 training set for 35 epochs. Moreover, 600 test images are used for the real-time evaluation of model performance at each epoch. The curves in Figure 3 show that the training loss of each model gradually decreases and then stabilizes, but the loss of the test set from EfficientNet b4 presents a decrease first and then increases trend. Based on the research [28], this indicates the growing overfitting problem for EfficientNet b4-b7 on the ISIC 2017 dataset, so we subsequently only focus on investigating the performance of EfficientNet b0-b3 on this dataset.

Further analysis indicates that EfficientNet b0-b3 help to obtain the best Avg. AUC of 0.904 (±0.017), 0.909 (±0.015), 0.903 (±0.021), and 0.902 (±0.013), respectively, and Table 3 also lists performances of other researches. According to the table, using ensembling multiple models or using external datasets also helps to achieve better performance, such as those by Xie et al. [29], Zhang et al. [30], and Matsunaga et al. [31]. Despite the negligible gap between best Díaz [32] using both “single-model” and “no-external-data,” the Avg. AUC achieved by EfficientNet b1 exceeds most ensembling models and those using external datasets. Obviously, the sensitivity for MEL of our approach (EfficientNet b1) is ahead of all the others, especially Díaz [32], and sensitivity for SK is second only to Matsunaga et al. [31].

**Table 3:** The performance of searched method on ISIC 2017 dataset (refer to https://challenge.isic-archive.com/leaderboards/2017 (Task 3: Lesion Diagnosis)) and derm_7pt dataset.

Team/authors	Use external data	Use ensemble models	Avg. AUC	BACC	Sensitivity (MEL, SK)	Specificity (MEL, SK)	Dataset
Xie et al. [29]	Yes	Yes	0.938	—	0.727, 0.844	0.915, 0.945	ISIC 2017
Zhang et al. [30]	Yes	No	0.917	—	0.878 (mean)	0.867 (mean)	
Matsunaga et al. [31]	Yes	Yes	0.911	0.831	0.735, 0.978	0.851, 0.773	
González et al. [32]	No	Yes	0.910	0.883	0.103, 0.178	0.998, 0.998	
Menegola et al. [33]	Yes	Yes	0.908	0.844	0.547, 0.356	0.950, 0.990	
Yu et al. [34]	Yes	Yes	0.897	—	—	—	
Yang et al. [35]	No	No	0.886	0.809	0.350,0.556	0.96, 0.976	
Our approach (EfficientNet b1)	No	No	0.909±0.015	0.821±0.025	0.743±0.016, 0.922±0.019	0.830±0.022, 0.856±0.019	
Kawahara et al. [16]	Yes	Yes	0.896	0.604	0.604 (mean)	0.91 (mean)	Derm7pt
Tudor et al. [36]	Yes	Yes	—	0.638	0.638 (mean)	0.926 (mean)	
Rodrigues et al. [37]	Yes	Yes	0.62	0.408	0.408 (mean)	0.710 (mean)	
Alzahrani et al. [38]	No	No	—	0.638	0.638 (mean)	0.702 (mean)	
Our approach (EfficientNet b0)	No	No	0.913±0.021	0.735±0.018	0.735±0.018 (mean)	0.92±0.020 (mean)	

The same training strategy was adopted in the generality verification on a smaller Derm7pt dataset consisting of both clinical and dermoscopic images. For the growing overfitting phenomenon of EfficientNet b4-b7 on a dataset of ISIC-skin 2017 scale, we just investigate the performance of EfficientNet b0-b3 on Derm7pt dataset. The best BACC of EfficientNet b0-b3 obtained is 0.735 (±0.018), 0.722 (±0.013), 0.721 (±0.019), and 0710 (±0.026), respectively, and all exceed the performance of the reports (Table 3) using ensembling models and external datasets.

**Figure 4:**
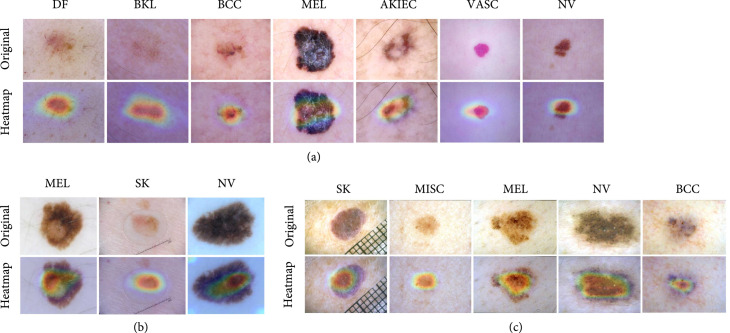
Heatmaps of lesions in different types in (a) HAM10000, (b) ISIC 2017, and (c) Derm7pt dataset.

As introduced previously, Grad-CAM++ is introduced to generate visual explanation heatmaps to highlight features affecting the prediction of models. We apply Grad-CAM++ to the best-performing models on HAM10000, ISIC 2017, and Derm7pt datasets, respectively. As shown in Figure 4, the heatmaps of typical lesions in each dataset highlight their related features very well, indicating that the model has indeed learned the ability to distinguish disease based on the corresponding lesion characteristics. In addition, this intuitive mark will be of high value in the presystemic screening of skin diseases. By obtaining whole-body skin pictures, completing the examination and positioning of suspected malignant lesions, the method will greatly reduce the workload of doctors and help them to perform more efficient diagnosis.

## 3. Discussion

AI-based diagnostic techniques can be potentially used to not only relieve dermatologists and dermatopathologists from time-consuming or repetitive tasks but also provide expert dermatologic care to rural populations, underserved communities, and regions of limited resources [8, 11]. Inspired by recent advances in computing technology and DCNN, various AI-based skin disease classifiers have been developed [12]. Although some of these classifiers have reported remarkable diagnostic accuracies equal or even superior to that of dermatology specialists, the outstanding performance is limited to the specific datasets and can hardly be replicated in general clinical data with consistent accuracy. Moreover, the outperformed DCNNs typically assemble multiple models that require significant computational resources or use external training sets inaccessible in the public domain, hindering their deployment in rural communities and regions of limited resources.

This project aims at developing a low-cost and high-performance data augmentation strategy that can be implemented in a low-complexity DCNN model for automatic skin cancer screening in rural communities. The proposed data augmentation strategy includes two consecutive stages of augmentation search and network search in a novel LCA search space. Compared with commonly used augmentation strategies such as AutoAugment [20], Fast AutoAugment [21], Population-Based Augmentation [22], and RandAugment, the size of LCA search space is only 60, representing a significant reduction of the search space and the computational costs. The performance of the proposed augmentation strategy is verified on the HAM10000 dataset using EfficientNet models. The best combination of the augmentation strategy and the DCNN model yields a BACC value of 0.853, ranking the first in the channel of “single-model and no-external-data” for task 3 of ISIC 2018 challenge. This result is even better than those of many ensemble models reported on the leaderboard. The generality of searched method is further verified on ISIC 2017 and Derm7pt datasets, in which the HAM10000-pretrained EfficientNets are fine-tuned based on both datasets. The EfficientNet b1 on ISIC 2017 helps to obtain the best Avg. AUC of 0.909 (±0.015), exceeding most ensembling models and those using external datasets. Meanwhile, EfficientNet b0 on smaller Derm7pt archives the best BACC of 0.735 (±0.018) ahead of all other related studies. Moreover, Grad-CAM++ is introduced to generate visual explanation heatmaps to validate and interpret the scientificity in the DCNN-based diagnosis. In addition to skin cancer classification, the proposed data augmentation strategy can be applied to other medical datasets in order to facilitate the development and deployment of low-cost, high-performance, and AI-based mobile devices for potentially automatic screening and potential medication guide of many diseases in rural communities and regions of limited resources.

Although the proposed augmentation strategy has a superior performance in the channel of “single-model and no-external-data,” its BACC is still behind the best performance on the ISIC 2018 challenge leaderboards by 0.4, and it also shows similar problems on the ISIC 2017 dataset. This gap is possibly due to insufficient representation of data augmentation or the possible overfitting of a single DCNN on the dataset. Future efforts will be made to update the augmentation search space in order to incorporate more invariances, add more data augmentation methods, and alleviate the issue of underrepresented patients in the medical image dataset [39]. In spite of the fact that our strategy only adopted EfficientNets as the baseline, we also test Regnets in other works and have achieved outstanding results too [40]. We also plan to test the proposed data enhancement method in combing with the self-attention-based networks such as Swin Transformer [41] or T2T-ViT [42]. In addition, data augmentation methods that have been validated in terms of fully supervised strategies will theoretically also work in self-supervised strategies and unsupervised strategies for the same kind of classification tasks [43], and we thereby plan to expand our proposed augmentation strategy in self-supervised and semi-supervised learning.

In addition, as shown in Figure 4, except for the function of highlighting features, the Grad-CAM++-based heatmaps also basically cover the scope of the lesion, which verifies the effectiveness and rationality of the network we trained. Moreover, the technology of Grad-cam++ has proved that it not only can be used in simple scenarios but also well localize category-related areas in complex scenes [25]. Although its ability to locate multiple lesions has not yet been verified, the resultant heatmaps are expected to be used to guide medical training.

## 4. Materials and Methods

### 4.1. Training and Search Strategies

**Figure 5:**
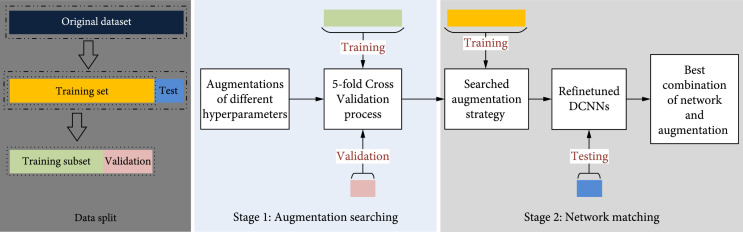
Work flow for the two-stage approach to search for the best combination of network and augmentation strategy.

Figure 5 illustrates the two-stage approach in search for the best combination of the augmentation and the network strategies for a specific clinical dataset. Prior to the search tasks, a novel data augmentation search space is defined, and the original dataset is divided into a training set and a test set. The training set is further divided into training and validation subsets. At stage one, the best augmentation strategy for the original dataset in the proposed data augmentation space is searched using 5-fold cross-validation. The search is based on the training set and the candidate DCNN models, where training and validation subset are randomly updated in different folds and the average of five best BACCs in the validation subsets is adopted as the screening criterion. At stage two, the DCNNs are refined by applying the best augmentation strategy using the full training set. After these two stages, the DCNN model with the best BACC on the test dataset and the best augmentation strategy will be preferentially recommended for subsequent clinical classification tasks.

In terms of computing cost to develop an intelligent diagnosis algorithm, it includes not only network training time and testing time but also the time to develop data enhancement strategies. While there is no obvious efficiency gap on the training and testing of similar networks with the premise of the same computing configuration, therefore the efficiency of developing diagnostic algorithms primarily depends on the computing cost of augmentation searching. Although the substantial search space of AutoAugment [20], Fast AutoAugment [21], Population-Based Augmentation [22], and RandAugment [23] will take an enormous amount of resources in researching for a clinical dataset, the transformations adopted have proven their effectiveness. Based on the above method, we propose a novel LCA augmentation search space that can be used extensively across different datasets for significant reduction of the search space. The proposed transformations (refer to Table S1) include not only the flip and scale changes and the AutoAugment liked strategies but also the randomly added Gaussian noise and the color tone shift [19, 20].

We believe that the synchronous application of color and shape policies will enhance the diversity of image and then improve the training outcome. Considering that there is a total of 12 different color strategies, the LCA search space is defined as an unordered set of 12 subpolicies. On the other hand, there are only 6 kinds of geometric change operations, and to ensure the same select probability of geometric operations, each of the geometric change operations is twice selected in the random match of subpolicies. Finally, one of the subpolicies shown in Table S2 will be randomly executed for each image in network training, and the execution probability of two different types of operations in the selected subpolicy is the same. Meanwhile, since the strategy ensures the same execute probability of color change operations and geometric change operations, we believe that the effects of recombination of substrategies are the same. We treat the augmentation search process as a discrete optimization problem [20]. In detail, the probability for executing each selected subpolicy is set as a ladder parameter in augmentation searching, and the corresponding execution magnitude is randomly determined within the specified range (refer to Table S1). Therefore, the only hyperparameter of the search space is the execution probability, and this greatly reduces the requirements for computational resources and thereby shortens the search time. Noticeably, the execution probability of two transformations in the selected subpolicy is the same, and the introduced notion of stochasticity into the augmentation policy enhances the robustness of the augmentation strategy.

### 4.2. Datasets and DCNNs

**Figure 6:**
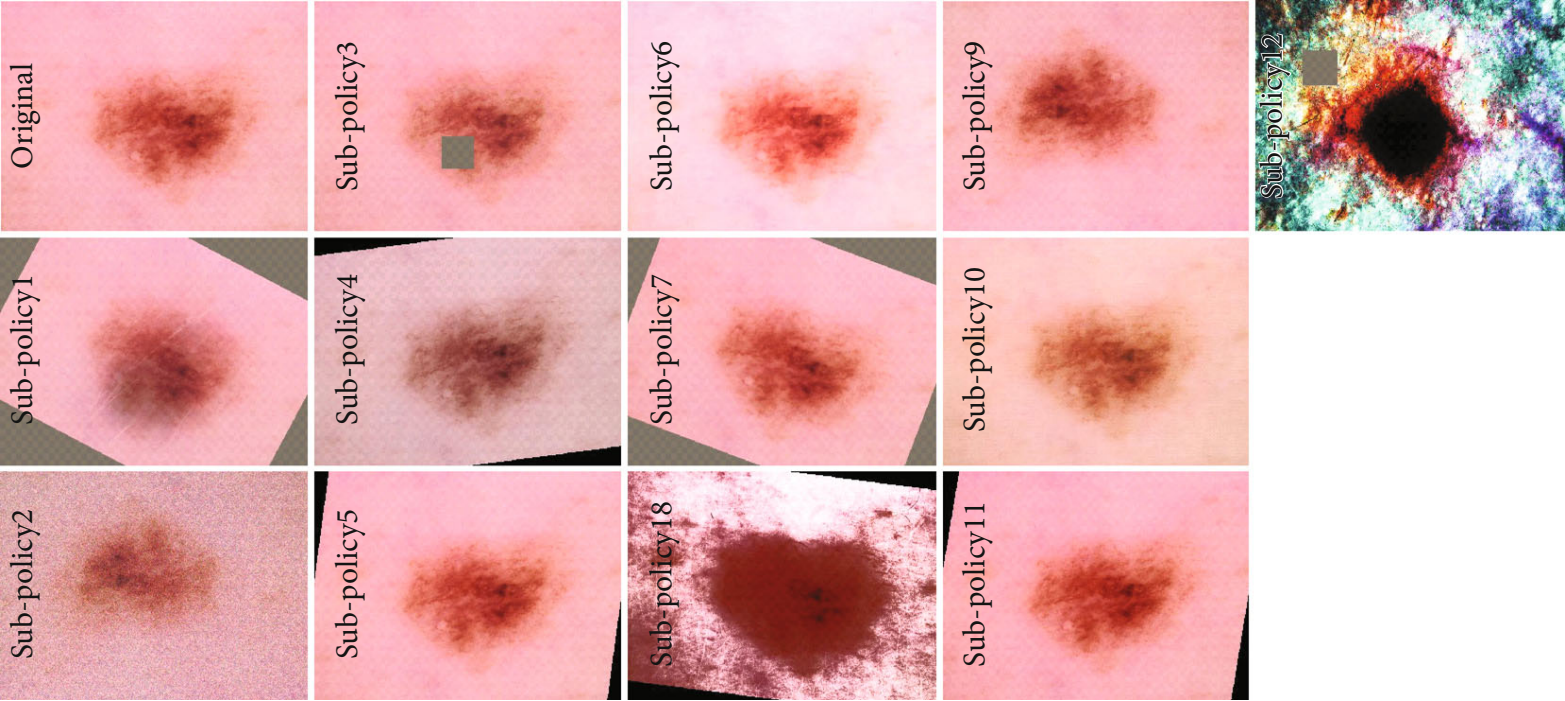
Representations of augmentation effects of different substrategies in the probability of 0.5.

The efficiency of the LCA-based data augmentation strategy is validated on a publicly accessible HAM10000 dataset and representative DCNNs. HAM10000 dataset contains 10015 skin lesion images. Out of them, 6705 are melanocytic nevi (NV); 1113 are melanoma (MEL); 1099 are benign keratosis-like lesion (BKL); 514 are basal cell carcinoma (BCC); 327 are actinic keratosis/Bowen’s diseases (AKIEC); 142 are vascular (VASC); and 115 are dermatofibroma (DF) [15]. Most of the HAM10000 images have the target lesions located at the center, and 53.30% of them are pathologically verified. HAM10000 is a publicly accessible dataset for the 2018 skin lesion analysis challenge organized by the International Skin Imaging Collaboration (ISIC) (https://www.isic-archive.com).Figure 6 shows a representative image from this dataset and a variety of images after applying different enhancement substrategies. The HAM10000 dataset is selected for testing our data augmentation strategy since its performance outcome can be easily compared with those of many other strategies published on the leaderboard.

Furthermore, the ISIC 2017 and Derm7pt are applied to evaluate the generality of the searched method. The ISIC 2017 dataset is a publicly available skin dermoscopy image dataset, consisting of 2000 training, 150 validations, and 600 test images screened for both privacy and quality assurance. Lesions in dermoscopy images are all paired with a gold standard (definitive) diagnosis, i.e., melanoma, nevus, and seborrheic keratosis. There are two binary classification subtasks for ISIC-skin 2017: melanoma classification (melanoma vs. others) and seborrheic keratosis classification (seborrheic keratosis vs. others). In addition, the Derm7pt dataset consists of 1011 images for each image modality (a total of 2022 images). The diagnosis consists of BCC, NV (blue, clark, combined, congenital, dermal, recurrent, and reed nevus), MEL (in situ, less than 0.76 mm, between 0.76 and1.5 mm, metastasis), miscellaneous (MISC) (dermatofibroma, lentigo, melanosis, miscellaneous, and vascular lesion), and SK. Alongside the images, relevant information as patient metadata and the 7-point checklist is provided. Here, just image data is used and 413, 203, and 395 cases are used to train, validate, and test data in the model finetune.

In terms of the DCNN models, we have evaluated the pretrained architectures and found that finely tuning a model trained on ImageNet performed significantly better than that trained from scratch. Previous studies also show better performance by using more recent architectures [26]. After comparing with several classic models such as Inception, ResNet, PolyNet, and DenseNet, we finally select the state-of-the-art EfficientNet model due to its better ImageNet accuracy that requires fewer parameters and FLOPs [24]. Another reason why we did not choose more networks is the lack of computing resources, and this is our original intention to develop low-cost data enhancement. Besides, since the lack of HAM10000 test data label and the modified limitation of maximum upload number for the official test, it is difficult to evaluate the performance, and this also hinders the test of more DCNN models.

### 4.3. Optimization and Verification of the Searching Strategy

Considering the important role that a training strategy plays in the final performance of DCNNs, we optimize the training strategy by implementing a 5-fold cross-validation procedure where the training set (HAM10000) is split into the training and the validation subsets following a ratio of 4 : 1 in each fold. The subset separation procedure ensures that the same lesion does not occur in both the training and the validation subsets and that the compositions of subsets vary randomly from different folds [26]. Especially, the strategy is finely tuned on the full HAM10000 dataset by applying the previously identified augmentation strategy at the stage of network searching, and the predicted classification results on the validation dataset have been uploaded to the ISIC challenge website for verification. Each image in the training set has an initial size of 600×450, and a randomly selected substrategy is executed following the defined probability. In this regard, the ladder probabilities are set as 0.1, 0.3, 0.5, 0.7, and 0.9. The image of 224×224 is randomly cropped from the augmented images and subsequently plug into the pretrained DCNNs with the modified output dimension of 7.

In the generality verification of the searched method on ISIC-skin 2017 dataset, HAM10000-pretrained EfficientNets combing the searched augmentation strategy are fine-tuned using training images and then evaluated using test images. Similarly, 413 images going through the searched augmentation strategy in the Derm7pt training group are applied to finetune the HAM10000-pretrained EfficientNets, and 395 test images are used to verify their performance. In the generality evaluation stage, images are equally cropped as 224×224 in random before plugging into the DCNNs with the modified output dimension.

In terms of the loss function, the following standard cross-entropy loss is used as a basis:

(1)p^i=eZi∑i=1C eZi,(2)L=−∑i=1C pi∗logp^i,

where $p_{i}$ is the ground-truth label of class i, $Z_{i}$ is the predicted score for class i, and C is the number of classes.

Since the seven classes in the original HAM10000 dataset are highly imbalanced, the multiclass weighted loss is implemented by adding an enhanced weight on the underrepresented classes, such as the highly underrepresented DF and VASC, to improve the overall performance of the trained DCNNs [26]. The multiclass weighted loss is updated by multiplying class-equilibrium matrix with the standard cross-entropy loss function, where the enhanced weight $w_{i}$ for class i corresponds to the inverse normalized class frequencies (equation (2)). The multiclass weighted loss is therefore updated as in equation (3):

(3)wi=N/ni,(4)L=−∑i=1C wi∗pi∗logp^i,

where N is the total number of samples and $n_{i}$ is the number of samples for class i.

The selection of other hyperparameters is more straightforward. First, we choose a starting learning rate of lr=0.001 and reduce it with a factor of λ=1/10 after 20 epochs. Then, we continue reducing the learning rate with the same factor at every 10 epochs and stop the optimization after 70 epochs. Finally, we select the best performing Adam as the optimizer for all the models. Considering that the same number of Graphics Processing Unit (GPU) carded is located for parallel searching tasks and that the feature map size of DCNNs increases proportionally with their parameters, using a uniform batch size may result in insufficient or waste of the computational resources. Therefore, we set the batch sizes for different models as model-specific values of 2^n^ (refer to Table S3 in Supplementary materials, where n is determined by the GPU capacity.

For the visual explanation stage, the gradient weights of the last convolutional layer feature maps for class i are first calculated [25]:

(5)αmnki=∂2Zi/∂Amnk22∂2Zi/∂Amnk2+∑m=1M∑n=1NAmnk∂3Zi/∂Amnk3,

where $Z_{i}$ is the predicted score for class i, $A^{k}$ is the k-th feature map of the last convolution layer, (m,n) and (M,N) are position and corresponding dimensions of the feature map $A^{k}$. Then, the weights $w_{k}^{i}\;$for the feature map $A^{k}$ and predicted class i is calculated as follows:

(6)wki=∑m=1M∑n=1Nαmnki∙relu∂Zi∂Amnk,

where relu function is used to get positive gradients. Finally, the visual explanation heatmap is generated by integrating the gradient weights and all K feature maps:

(7)Lmni=relu∑k=1Kwki∙Amnk.

All the training and testing tasks are performed on NVIDIA GeForce GTX 2080Ti graphics cards using the popular frameworks PyTorch [44] and the PyTorch pretrained models library. In comparison, the EfficientNet models are also trained by applying the identified AutoAugment strategy on ImageNet [20] and by applying the General Augmentation strategy that composes only random flip and color jitter.

### 4.4. Metrics for Cost and Performance Evaluation

The computational cost of the proposed strategy is evaluated by the search space size, defined as the order of magnitude for the number of possible transformations [23]. The diagnostic performance of the proposed strategy is evaluated by the balanced accuracy (BACC) across the seven classes, equivalent to the average recall or sensitivity [26]. A multicrop evaluation strategy is used for the generation of the final predictions, and the performance is generally better after averaging is applied [26]. Specifically, 16 regions of interests (ROIs) with the size of 224×224 were equidistantly cropped from the upper left corner to the lower right corner of each unscaled image, and an average across all the predictions is used as a benchmark for final prediction.

The following metrics are used to evaluate the prediction performance on class *i*:

(8)Precisioni=TPiTPi+FPi,Sensitivityi=TPiFNi+TPi,Specificityi=TNiTNi+FPi,Accuracyi=TNi+TPiTNi+FPi+FNi+TPi,

where $TP_{i}$ is the number of true positive cases in class i, $FN_{i}$ is the number of false negative cases in class i, $TN_{i}$ is the number of true negative cases, and $FP_{i}$ is number of false positive cases, all in class i.

The key metric of BACC for ISIC 2018 challenge (C=7) and Derm7pt (C=5) is defined in equation (9), which is also used as the metric for our preliminary performance evaluation and hyperparameter tuning.

(9)BACC=1C∑i=1CTPiTPi+FNi =1C∑i=1C Sensitivityi 

The key metric for ISIC-skin 2017 is AUC defined as the entire two-dimensional area underneath the entire receiver operating characteristic curve from (0,0) to (1,1).

## Data Availability

The datasets of HAM10000 and ISIC 2017 used to support the findings of this study are publicly accessible on the International Skin Imaging Collaboration (ISIC) (https://www.isic-archive.com); the Derm7pt data used to support this study is available at doi:10.1109/JBHI.2018.2824327.

## Supplementary Materials

Supplementary Materials Supplementary Information containing supplementary Table S1-4: list of all image transformations used in the Low-Cost-Augment, list of all the subpolicies in LCA-based augmentation, batch size used during training for different EfficientNets, and detailed performance of the best model on the official test data.

## References

[1] D. Seth, K. Cheldize, D. Brown, and E. F. Freeman, “Global burden of skin disease: inequities and innovations,” *Current Dermatology Reports*, vol. 6, no. 3, pp. 204–210, 2017 29226027 10.1007/s13671-017-0192-7 PMC5718374

[2] A. C. Society *Cancer Facts & Figures*, The Society, 2021

[3] M. A. Linares, A. Zakaria, and P. Nizran, “Skin cancer,” *Primary Care*, vol. 42, no. 4, pp. 645–659, 2015 26612377 10.1016/j.pop.2015.07.006

[4] S. Machlin, K. Carper, and D. Kashihara *Health Care Expenditures for Non-Melanoma Skin Cancer among Adults*, Agency for Healthcare Research and Quality, Rockville, 2011, https://meps.ahrq.gov/data_files/publications/st345/stat345.pdf.

[5] E. Akar, O. Marques, W. Andrews, and B. Furht, “Cloud-based skin lesion diagnosis system using convolutional neural networks,” *Intelligent Computing. CompCom 2019*, K. Arai, R. Bhatia, and S. Kapoor, Eds., Springer, Cham, vol. 997, Advances in Intelligent Systems and Computing, pp. 982–1000

[6] T. P. Habif, M. S. Chapman, J. G. Dinulos, and K. A. Zug *Skin Disease e-Book: Diagnosis and Treatment*, Elsevier Health Sciences, 2017

[7] C. Rosendahl, P. Tschandl, A. Cameron, and H. Kittler, “Diagnostic accuracy of dermatoscopy for melanocytic and nonmelanocytic pigmented lesions,” *Journal of the American Academy of Dermatology*, vol. 64, no. 6, pp. 1068–1073, 2011 21440329 10.1016/j.jaad.2010.03.039

[8] O. Abuzaghleh, B. D. Barkana, and M. Faezipour, “Noninvasive real-time automated skin lesion analysis system for melanoma early detection and prevention,” *IEEE Journal of Translational Engineering in Health and Medicine*, vol. 3, article 2900310, 2015 10.1109/JTEHM.2015.2419612 PMC4848099 27170906

[9] R. B. Oliveira, M. E. Filho, Z. Ma, J. P. Papa, A. S. Pereira, and J. M. Tavares, “Computational methods for the image segmentation of pigmented skin lesions: a review,” *Computer Methods and Programs in Biomedicine*, vol. 131, pp. 127–141, 2016 27265054 10.1016/j.cmpb.2016.03.032

[10] M. A. Taufiq, N. Hameed, A. Anjum, and F. Hameed, “m-Skin Doctor: a mobile enabled system for early melanoma skin cancer detection using support vector machine,” *eHealth 360°*, Springer, Cham, vol. 181, Lecture Notes of the Institute for Computer Sciences, Social Informatics and Telecommunications Engineering, pp. 468–475, 2017

[11] S. S. Han, M. S. Kim, W. Lim, G. H. Park, I. Park, and S. E. Chang, “Classification of the clinical images for benign and malignant cutaneous tumors using a deep learning algorithm,” *The Journal of Investigative Dermatology*, vol. 138, no. 7, pp. 1529–1538, 2018 29428356 10.1016/j.jid.2018.01.028

[12] N. Hameed, A. M. Shabut, F. Hameed, S. Cirstea, S. Harriet, and M. A. Hossain, “Mobile-based skin lesions classification using convolution neural network,” *Annals of Emerging Technologies in Computing*, vol. 4, no. 2, pp. 26–37, 2020

[13] J. Deng, W. Dong, R. Socher, L.-J. Li, K. Li, and L. Fei-Fei, “Imagenet: a large-scale hierarchical image database,” in *2009 IEEE Conference on Computer Vision and Pattern Recognition*, Miami, FL, USA, 2009, pp. 248–255

[14] T. Mendonça, P. M. Ferreira, J. S. Marques, A. R. Marcal, and J. Rozeira, “PH^2^-A dermoscopic image database for research and benchmarking,” in *2013 35th Annual International Conference of the IEEE Engineering in Medicine and Biology Society (EMBC)*, Osaka, Japan, 2013, pp. 5437–5440 10.1109/EMBC.2013.6610779 24110966

[15] P. Tschandl, C. Rosendahl, and H. Kittler, “The HAM10000 dataset, a large collection of multi-source dermatoscopic images of common pigmented skin lesions,” *Scientific Data*, vol. 5, no. 1, article 180161, 2018 10.1038/sdata.2018.161 PMC6091241 30106392

[16] J. Kawahara, S. Daneshvar, G. Argenziano, and G. Hamarneh, “Seven-point checklist and skin lesion classification using multitask multimodal neural nets,” *IEEE Journal of Biomedical and Health Informatics*, vol. 23, no. 2, pp. 538–546, 2019 10.1109/JBHI.2018.2824327 29993994

[17] C. Elkan *Predictive Analytics and Data Mining*, University of California, 2013

[18] A. Nozdryn-Plotnicki, J. Yap, and W. Yolland *Ensembling convolutional neural networks for skin cancer classification*, International Skin Imaging Collaboration (ISIC) Challenge on Skin Image Analysis for Melanoma Detection. MICCAI, 2018

[19] I. Sato, H. Nishimura, and K. Yokoi, “Apac: augmented pattern classification with neural networks,”, 2015, https://arxiv.org/abs/1505.03229.

[20] E. D. Cubuk, B. Zoph, D. Mane, V. Vasudevan, and Q. V. Le, “Autoaugment: learning augmentation strategies from data,” in *Proceedings of the IEEE conference on computer vision and pattern recognition*, Long Beach, CA, USA, 2019, pp. 113–123

[21] S. Lim, I. Kim, T. Kim, C. Kim, and S. Kim, “Fast autoaugment,” *Advances in Neural Information Processing Systems*, pp. 6665–6675, 2019, https://arxiv.org/abs/1905.00397.

[22] D. Ho, E. Liang, X. Chen, I. Stoica, and P. Abbeel, “Population based augmentation: efficient learning of augmentation policy schedules,” in *International Conference on Machine Learning*, Long Beach, CA, USA, 2019, pp. 2731–2741

[23] E. D. Cubuk, B. Zoph, J. Shlens, and Q. V. Le, “Randaugment: practical automated data augmentation with a reduced search space,” in *Proceedings of the IEEE/CVF Conference on Computer Vision and Pattern Recognition Workshops*, Seattle, USA, 2020, pp. 702–703

[24] M. Tan, and Q. Le, “Efficientnet: rethinking model scaling for convolutional neural networks,” in *International Conference on Machine Learning*, Long Beach, California, 2019, pp. 6105–6114

[25] A. Chattopadhay, A. Sarkar, P. Howlader, and V. N. Balasubramanian, “Grad-cam++: generalized gradient-based visual explanations for deep convolutional networks,” in *2018 IEEE Winter Conference on Applications of Computer Vision (WACV)*, Lake Tahoe, NV, USA, 2018, pp. 839–847

[26] N. Gessert, T. Sentker, F. Madesta, R. Schmitz, H. Kniep, I. Baltruschat, R. Werner, and A. Schlaefer, “Skin lesion diagnosis using ensembles, unscaled multi-crop evaluation and loss weighting,”, 2018, https://arxiv.org/abs/1808.01694.

[27] A. Mahbod, G. Schaefer, C. Wang, G. Dorffner, R. Ecker, and I. Ellinger, “Transfer learning using a multi-scale and multi-network ensemble for skin lesion classification,” *Computer Methods and Programs in Biomedicine*, vol. 193, p. 105475, 2020 32268255 10.1016/j.cmpb.2020.105475

[28] M. Cogswell, F. Ahmed, R. Girshick, L. Zitnick, and D. Batra, “Reducing overfitting in deep networks by decorrelating representations,”, 2015, https://arxiv.org/abs/1511.06068.

[29] Y. Xie, J. Zhang, Y. Xia, and C. Shen, “A mutual bootstrapping model for automated skin lesion segmentation and classification,” *IEEE Transactions on Medical Imaging*, vol. 39, no. 7, pp. 2482–2493, 2020 32070946 10.1109/TMI.2020.2972964

[30] J. Zhang, Y. Xie, Y. Xia, and C. Shen, “Attention residual learning for skin lesion classification,” *IEEE Transactions on Medical Imaging*, vol. 38, no. 9, pp. 2092–2103, 2019 30668469 10.1109/TMI.2019.2893944

[31] K. Matsunaga, A. Hamada, A. Minagawa, and H. Koga, “Image classification of melanoma, nevus and seborrheic keratosis by deep neural network ensemble,”, 2017, https://arxiv.org/abs/1703.03108.

[32] I. G. Díaz, “Incorporating the knowledge of dermatologists to convolutional neural networks for the diagnosis of skin lesions,”, 2017, https://arxiv.org/abs/1703.01976. 10.1109/JBHI.2018.2806962 29994788

[33] A. Menegola, J. Tavares, M. Fornaciali, L. T. Li, S. Avila, and E. Valle, “RECOD titans at ISIC challenge 2017,”, 2017, https://arxiv.org/abs/1703.04819.

[34] Z. Yu, F. Jiang, F. Zhou, X. He, D. Ni, S. Chen, T. Wang, and B. Lei, “Convolutional descriptors aggregation via cross-net for skin lesion recognition,” *Applied Soft Computing*, vol. 92, p. 106281, 2020

[35] X. Yang, Z. Zeng, S. Y. Yeo, C. Tan, H. L. Tey, and Y. Su, “A novel multi-task deep learning model for skin lesion segmentation and classification,”, 2017, https://arxiv.org/abs/1703.01025.

[36] T. Nedelcu, M. Vasconcelos, and A. Carreiro, “Multi-dataset training for skin lesion classification on multimodal and multitask deep learning,” in *Proceedings of the 6th World Congress on Electrical Engineering and Computer Systems and Sciences (EECSS’20)*, Prague, Czech Republic, 2020, pp. 13–15

[37] J. F. Rodrigues, B. Brandoli, and S. Amer-Yahia, “DermaDL: advanced convolutional neural networks for automated melanoma detection,” in *2020 IEEE 33rd International Symposium on Computer-Based Medical Systems (CBMS)*, Rochester, MN, USA, 2020, pp. 504–509

[38] S. Alzahrani, W. Al-Nuaimy, and B. Al-Bander, “Seven-point checklist with convolutional neural networks for melanoma diagnosis,” in *2019 8th European Workshop on Visual Information Processing (EUVIP)*, Roma, Italy, 2019, pp. 211–216

[39] Y. Jiang, J. H. Xiong, H. Y. Li, X. H. Yang, W. T. Yu, M. Gao, X. Zhao, Y. P. Ma, W. Zhang, Y. F. Guan, H. Gu, and J. F. Sun, “Recognizing basal cell carcinoma on smartphone-captured digital histopathology images with a deep neural network,” *British Journal of Dermatology*, vol. 182, no. 3, pp. 754–762, 2020 31017653 10.1111/bjd.18026

[40] P. Yao, S. Shen, M. Xu, P. Liu, F. Zhang, J. Xing, P. Shao, B. Kaffenberger, and R. X. Xu, “Single model deep learning on imbalanced small datasets for skin lesion classification,” *IEEE Transactions on Medical Imaging*, 2021 10.1109/TMI.2021.3136682 34928791

[41] Z. Liu, Y. Lin, Y. Cao, H. Hu, Y. Wei, Z. Zhang, S. Lin, and B. Guo, “Swin transformer: hierarchical vision transformer using shifted windows,”, 2021, https://arxiv.org/abs/2103.14030.

[42] L. Yuan, Y. Chen, T. Wang, W. Yu, Y. Shi, Z. Jiang, F. E. H. Tay, J. Feng, and S. Yan, “Tokens-to-token vit: training vision transformers from scratch on imagenet,”, 2021, https://arxiv.org/abs/2101.11986.

[43] Q. Xie, Z. Dai, E. Hovy, M.-T. Luong, and Q. V. Le, “Unsupervised data augmentation for consistency training,” *Advances in Neural Information Processing Systems*, vol. 33, pp. 6256–6268, 2019

[44] A. Paszke, S. Gross, S. Chintala, G. Chanan, E. Yang, Z. DeVito, Z. Lin, A. Desmaison, L. Antiga, and A. Lerer, “Automatic differentiation in PyTorch,” *In NIPS Workshop*, 2017

