# Erratum to “A Low-Cost High-Performance Data Augmentation for Deep Learning-Based Skin Lesion Classification”

**DOI:** 10.34133/bmef.0011

**Published:** 2023-03-31

**Authors:** Shuwei Shen, Mengjuan Xu, Fan Zhang, Pengfei Shao, Honghong Liu, Liang Xu, Chi Zhang, Peng Liu, Peng Yao, Ronald X. Xu

**Affiliations:** ^1^ First Affiliated Hospital, University of Science and Technology of China, Hefei 230031, China.; ^2^ Suzhou Institute for Advanced Research, University of Science and Technology of China, Suzhou 215000, China.; ^3^ Department of Precision Machinery and Precision Instrumentation, University of Science and Technology of China, Hefei 230026, China.

In the Research Article “A Low-Cost High-Performance Data Augmentation for Deep Learning-Based Skin Lesion Classification,” Zhihong Zhang was erroneously listed as an author. The PDF and HTML (full text) have been corrected.
