# Supplementary material for: A Low-Cost High-Performance Data Augmentation for Deep Learning-Based Skin Lesion Classification
Source: BME Front. 2022 Apr 26;2022:9765307. doi: 10.34133/2022/9765307 (PMC10521644; doi:10.34133/2022/9765307)
Supplement: Supplementary Materials — Supplementary Information containing supplementary Table S1-4: list of all image transformations used in the Low-Cost-Augment, list of all the subpolicies in LCA-based augmentation, batch size used during training for different EfficientNets, and detailed performance of the best model on the official test data. [file 9765307.f1.docx]

SUPPLEMENTARY MATERIALS

Supplementary Information containing supplementary Table S1-4: List of all image transformations used in the Low-Cost-Augment, List of all the sub-policies in LCA-based augmentation, Batch-size used during training for different EfficientNets, The detailed performance of the best model on the official test data. (Supplementary Materials)

TABLE S1: List of all image transformations used in the Low-Cost-Augment. List of all image transformations used in the Low-Cost-Augment. Additionally, the values of magnitude that can be applied by the controller during the search for each operation are shown in the second column. Some transformations do not use the magnitude information (e.g. Invert and Equalize).

| **Operation name** | **Range of magnitude** | **Description** |
| --- | --- | --- |
| Sample_Pairing | [0, 0.4] | Linearly add the image with another image (randomly selected from the same batch) with weight magnitude, without changing the label. |
| Gaussian_noise | [0, 0.4] | Add random Gaussian noise to the image with rate magnitude. |
| SolarizeAdd | [1, 110] | For each pixel in the image that is less than 128, add an additional amount to it decided by the magnitude. |
| Color | [0.1, 1.9] | Adjust the color balance of the image, in a manner similar to the controls on a color TV set. A magnitude=0 gives a black & white image, whereas magnitude=1 gives the original image. |
| Contrast | [0.1, 1.9] | Control the contrast of the image. A magnitude=0 gives a gray image, whereas magnitude=1 gives the original image |
| Brightness | [0.1, 1.9] | Adjust the brightness of the image. A magnitude=0 gives a black image, whereas magnitude=1 gives the original image. |
| Sharpness | [0.1, 1.9] | Adjust the sharpness of the image. A magnitude=0 gives a blurred image, whereas magnitude=1 gives the original image. |
| Color_shift | [-20, 20] | Add a random magnitude value to R, G, and B channels respectively |
| Equalize_YUV |  | Equalize the histogram of each YUV channel after transferring image into YUV color spaces. |
| Equalize |  | Equalize the histogram of each RGB channel of image respectively |
| Posterize | [4, 8] | Reduce the number of bits for each pixel to magnitude bits. |
| AutoContrast |  | Maximize the image contrast, by making the darkest pixel black and lightest pixel white |
| Rotate | [-30, 30] | Rotate the image magnitude degrees. |
| Flip |  | Flip image randomly in horizontal or vertical axis |
| Cutout | [0, 60] | Set a random square patch of side-length magnitude pixels to gray. |
| ShearX(Y) | [-0.3, 0.3] | Shear the image along the horizontal (vertical) axis with rate magnitude. |
| Scale | [0.6, 1.4] | Randomly scale the picture proportionally with rate magnitude. |

TABLE S2: List of all the sub-policies in LCA-based augmentation. (Op: operation, P: probability)

| **Policy** | **Op 1** | **Op 2** | **P** | **Policy** | **Op 1** | **Op 2** | **P** |
| --- | --- | --- | --- | --- | --- | --- | --- |
| 1 | Sample_Pairing | Rotate | [0, 1] | 7 | Sharpness | Rotate | [0, 1] |
| 2 | Gaussian_noise | Flip | [0, 1] | 8 | Color_shift | Scale | [0, 1] |
| 3 | SolarizeAdd | Cutout | [0, 1] | 9 | Equalize_YUV | ShearX | [0, 1] |
| 4 | Color | ShearX | [0, 1] | 10 | Posterize | ShearY | [0, 1] |
| 5 | Contrast | ShearY | [0, 1] | 11 | AutoContrast | Flip | [0, 1] |
| 6 | Brightness | Scale | [0, 1] | 12 | Equalize | Cutout | [0, 1] |

TABLE S3: Batch-size used during training for different EfficientNets

| **DCNNs** | **Batch-size** | **DCNNs** | **Batch-size** | **DCNNs** | **Batch-size** |
| --- | --- | --- | --- | --- | --- |
| EfficientNet b0 | 64 | EfficientNet b3 | 32 | EfficientNet b6 | 16 |
| EfficientNet b1 | 64 | EfficientNet b4 | 32 | EfficientNet b7 | 16 |
| EfficientNet b2 | 64 | EfficientNet b5 | 16 |  |  |

TABLE S4: The detailed performance of the best model on the official test data. (AUC: Area under the receiver operating characteristic curve).

| **Category Metrics** | **Mean Value** | **Diagnosis Category** | | | | | | | |
| --- | --- | --- | --- | --- | --- | --- | --- | --- | --- |
|  |  | **MEL** | **NV** | **BCC** | **AKIEC** | **BKL** | **DF** | **VASC** |  |
| AUC | 0.975 | 0.943 | 0.970 | 0.989 | 0.988 | 0.968 | 0.972 | 0.995 |  |
| Average Precision | 0.870 | 0.785 | 0.981 | 0.897 | 0.826 | 0.873 | 0.813 | 0.915 |  |
| Accuracy | 0.958 | 0.925 | 0.902 | 0.980 | 0.979 | 0.938 | 0.990 | 0.991 |  |
| Sensitivity | 0.853 | 0.789 | 0.869 | 0.882 | 0.907 | 0.811 | 0.795 | 0.914 |  |
| Specificity | 0.979 | 0.958 | 0.962 | 0.993 | 0.981 | 0.968 | 0.996 | 0.993 |  |
